# Partner notification service utilization and associated factors among clients attending anti-retroviral therapy clinics of public health facilities in Gimbi Town, West Ethiopia, 2023: a facility-based mixed-method cross-sectional study

**DOI:** 10.1186/s12889-024-18196-4

**Published:** 2024-03-04

**Authors:** Habtamu Oljira, Abiriham Rata, Befirdu Mulatu, Tesfaye Abera

**Affiliations:** 1 Department of Biomedical Science, School of Medicine, Wallaga University, Nekemte, Oromia Ethiopia; 2 Nekemte Blood Bank, Oromia Health Beuareu, Nekemte, Oromia Ethiopia; 3 Department of Public Health, Institute of Health Science, Wallaga University, Nekemte, Oromia Ethiopia; 4 Department of Nursing, School of Nursing and Midwifery, Wallaga University, Nekemte, Oromia Ethiopia

**Keywords:** Partner notification, Human immunodeficiency virus Testing, Sexual Partner

## Abstract

**Background:**

Partner Notification Service is among the strategies used to conduct targeted Human Immunodeficiency Virus Testing Service by obtaining information about sexual contacts of index clients to refer for testing. But most people living with Human Immunodeficiency Virus are still unaware of their status, including Ethiopia. Limited studies are available on the magnitude of partner notification service utilization and associated factors in Ethiopia.

**Objective:**

The aim of this study was to assess the magnitude of partner notification service utilization and associated factors among people living with Human Immunodeficiency Virus attending anti-retroviral therapy clinics of public health facilities in Gimbi town, West Ethiopia.

**Methods:**

A facility-based mixed-method cross-sectional study design was used. Total of 455 study participants were selected by systematic random sampling for quantitative data and health workers were purposively selected for qualitative data until saturation of ideas was reached. The study was conducted from December 1, 2022 to January 30, 2023. Structured questionnaires and key informant interview guides were used for data collection. Quantitative data were analyzed using Statistical Package for Social Science version 25. Open code 4.02 software was used for qualitative data analysis. Frequencies and proportions were used to summarize descriptive statistics. Bivariable and multivariable logistic regression was used to identify associated factors then variables with a *p* value < 0.05 were declared to have an association with the dependent variable.

**Result:**

Exactly 298 (65.5%) of the study participants were notified their HIV status to their sexual partners. Factors associated with Partner Notification Service Utilization were depression AOR: 0.12 (95% CI: 0.07, 0.20), urban settlers AOR: 2.21 (95% CI: 1.2, 3.83), fear of support loss AOR: 0.24 (95% CI: 0.14, 0.40) and intimate partner violence AOR: 0.55 (95% CI: 0.31, 0.97). From qualitative part of this study, factors associated to Partner Notification service utilization were fear of stigma, discrimination and fear of divorce.

**Conclusion:**

Two-third of the study participants were utilized partner notification service, and efforts are important to prevent depression and intimate partner violence. Local government bodies and stakeholders should implement economic strengthening and strategies to address the rural community for HIV/AIDS prevention. Promotion of supportive and inclusive environment for PLHIV should also considered as way to increase PNS utilization.

## Background

Human Immunodeficiency Virus (HIV) Partner Notification Services (PNS) is an HIV prevention strategy in which a trained provider asks people diagnosed with HIV to voluntarily provide information about their sexual partners and then attempts are made to contact them for HIV testing [1, 2]. This has the potential to identify people with undiagnosed HIV infection which will lessen transmission of the case and enable HIV-negative clients to choose the best strategy to remain uninfected [2, 3].

The World Health Organization (WHO) developed guidelines in 2016 advocating the Human Immunodeficiency Virus Testing Service (HTS) for couples and partners, including mutual disclosure support with a special focus on testing the sexual partners of people diagnosed with HIV infection in all epidemiological settings [4]. According to Ethiopia’s current national consolidated guidelines on Comprehensive HIV Prevention, Care and Support, sexual partners of index clients with HIV and sexually transmitted infection (STI) diagnoses should be notified as a standard part of clinical practice and patient management [3].

When compared to the WHO recommendation statement that all sexual partners of HIV positive individuals should be offered partner notification services, evidence on the proportion of elicitation and notification of sexual partners of HIV-positive index clients in different epidemiologic settings indicates a very low to averagely acceptable level [5–7]. This underutilization of partner notification service shares the consequences of leaving 16% of people living with human immunodeficiency virus (PLHIV) unaware of their HIV status globally [4, 6]. According to a study conducted in 2019 to explore trends and spatial distribution of HIV in Ethiopia, there were more than half a million (729,089) PLHIV, 21,606 new HIV infections (of which 21% were unaware of their HIV status) and 10,960 deaths in 2018 [8]. It has a varied geographical distribution ranging from 0.1% in Somale regional state to 4.8% in Gambella regional state [8, 9].

Some studies across the world have tried to identify contributory factors for this underutilization of partner notification services, such as age of index client, marital status, gender, white race, intimate partner violence, financial support, fear of stigma, couple HIV testing and counseling and number of children [6, 10, 11]. Additionally, abandoning informed consent, violating privacy and confidentiality, inaccessibility of health care service, undeveloped health service infrastructure, adherence to anti-retroviral therapy (ART), depression, social isolation, criminalization of drug use and verbal or emotional abuse were factors associated with partner notification service utilization. However, there was no study on this particular issue in Ethiopia to the knowledge of investigators [5, 7, 12–17].

The WHO strongly recommends using comprehensive HIV testing strategies such as community based, home based, mobile testing and partner notification services to meet the first 90 goals of reaching a larger and more diverse population earlier in the course of infection to address the undiagnosed portion of HIV patients [1, 4]. However, the utilization of partner notification services cannot bring the intended result across the world uniformly [4]. To achieve the first 90 targets and reach out to 21% of PLHIV unaware of their status, the Ethiopian Ministry of Health has adapted and implemented new ways that can increase the efficiency and coverage of testing needed, one of which is HIV partner notification [18]. This has the potential to improve coverage as well as identify persons with HIV infection who have not been diagnosed [18]. However, the magnitude of partner notification service utilization and associated factors has not yet been studied and is well known to the investigator’s knowledge in our country.

This study therefore provided evidence on the extent to which partner notification service was utilized for HIV testing and what factors influence the service delivery in the study area.

## Methods

### Study Setting and participants

A facility based convergent design of mixed method cross-sectional study was conducted at the ART clinics of public health facilities in Gimbi town from December 1, 2022, to January 30, 2023. Gimbi town has three public health facilities, namely, Gimbi General Hospital, Gimbi Adventist General Hospital and Gimbi Health Center, which are currently providing comprehensive clinical services, including ART. The town is the capital of the West Wollega Zone and is found in the west direction from Finfinne/Addis Ababa at a distance of 420 km. The total population of the town is 53,940, of which 26,430 are males and 27,510 are females. Currently, there are 1355 adult PLHIV (664 males and 691 females) on ART services in the three health facilities where the study was conducted.

### Sample size determination and sampling technique

For quantitative, the sample size was calculated using a single population proportion formula by considering 37.6% of HIV status disclosure among PLHIV on ART follow-up at Jimma University Specialized Hospital, which was conducted by a facility-based cross-sectional study design, 95% confidence interval and 5% margin of error [19]. In addition, the sample size was also determined using Epi-info version 7 by taking factors that have statistically significant associations in previously related studies performed on similar issues by considering 80% power, 95% confidence intervals, outcomes in the unexposed group and outcomes in the exposed group. Based on the above criteria, the largest sample size was determined to be 417 from sex as a factor that has statistically significant association with PNS in previous study. Finally, after adding none response rate of 10%, sample needed for this study was determined to be 459.

For qualitative, purposive sampling was used to select participants living with HIV providing adherence support services for in-depth interviews and ART service providers for key informant interviews at the respective health facility ART units until saturation of the idea was reached. The key informant interview participants were health care workers who are in charge of providing HIV care, treatment and support at respective health facilities.

From 1355, the total number of adult index clients currently on ART at three public health facilities in Gimbi town where ART service is being given, samples representing each health facility was calculated using proportional allocation. Finally, a systematic random sampling technique was used to select 455 study participants when they came to health facilities for HIV care and support. Initially, the minimum daily index client flow for HIV care and support was determined to be an average of twelve [20] to fifteen [12] per day. Then, study participants were selected based on the sequence of coming to the health facility by calculating the interval, which is every three observations. For qualitative study participants, health care workers providing ART service and adherence supporters at respective health facilities were selected purposively until saturation of ideas on interview simultaneously with quantitative data collection. All index clients on follow-up of ART service aged 18 and above were included in the study while index clients less than 18 years old age and seriously ill were excluded.

### Data collection tool and procedure

The survey tool used in this study to gather the relevant data was adapted from earlier studies on a related topic that were conducted in English. Then, it was translated into Afan Oromo by an expert in both languages and after reorganizing, it was translated back into English for accuracy and consistency [1, 21–25]. Experts in both languages had also checked for consistency of meaning and use of appropriate words of the Afan Oromo translation. An interviewer-administered questionnaire was used to interview study participants at the three health facilities. Quantitative data were collected using a structured questionnaire prepared in English and translated to Afan Oromo to collect information on sociodemographic characteristics, experience of PLHIV in partner notification service utilization, psychological factors and health system factors related to partner notification for HIV testing, while qualitative data were collected using a semistructured interview guide. Pretest was conducted before actual data collection on 5%(23) of 459 sample size to test the questioner. Data collectors were ART data clerks at each health facility under the follow-up of supervisors assigned to execute this function. Quantitative data was collected by face to face interview of study participants who came for ART service during the study period based on the calculated interval for the selection of study participants depending on the sequence of coming to the health facility. Research assistants used interview guides and mobile cell phones to record responses from selected ART providers and adherence supporters at each health facility to collect qualitative data.

### Data analysis

Quantitative data were coded and entered into Epi data version 4.2 and exported to Statistical Package for Social Science (SPSS) version 25 for analysis. Descriptive statistics were computed and presented using tables and charts. Model goodness of fit was assessed by using the Hosmer and Lemeshow test. Multicollinearity between independent variables was checked by the variance inflation factor. Bivariate logistic regression was executed, and variables with *p* < 0.25 were fitted to the final multivariable logistic regression to adjust for potential confounders. In the final model, variables with a *P* value < 0.05 and adjusted odds ratio (AOR) of 95% confidence interval was considered to declare statistical significance and the strength of association.

Simultaneously with quantitative data analysis, electronically recorded data and the detailed notes transcribed were translated into English by the principal investigator to import into an open code for facilitation of coding. Based on key concepts, some codes were developed, and care was taken to ensure that each respondent’s accuracy of meaning. Qualitative data were thematically analyzed using Open code version 4.02 software after generating thematic areas for each data point.

### Ethical considerations

The ethical approval letter of permission was obtained from the Ethical Review Committee of Wallaga University, Institute of Health Sciences before conducting the study. Then, a formal letter was written to Gimbi Health Center, Gimbi General Hospital and Gimbi Adventist Hospital. The letters were submitted to these health facilities to obtain permission for the study. Informed verbal consent was obtained from study participant came for HIV treatment, care and support service after informing them all the purpose and benefits of the study. No study participant under eighteen years of age included in the study, so there was no need to obtain consent from parents or legal guardians. The verbal informed consent was approved by the Ethical Review Committee of the university. Data were analyzed as group data and not individually. No names were recorded or linked to the results of the survey.

## Results

Out of 459 samples needed for the study, 455 HIV positive adult patients who are currently on follow-up at ART clinics of public health facilities in Gimbi town were interviewed, yielding a 99.1% response rate. The mean age of the study participants was 36.21 with a standard deviation of ±6.97 years. Among the study participants, 249 (54.7%) were females. From all study participants, 378 (83.1%) of them were from the Oromo ethnic group, followed by Amhara 68 (14.9%) (Table 1).

**Table 1:** Sociodemographic Characteristics of ART Clinic Attendants at Gimbi town Public Health Facilities (*n* = 455), January 2023

Characteristics	Category	Frequency	Percent
Age group	20–24	5	1.1
	25–29	79	17.4
	30–34	128	28.1
	35–39	88	19.3
	40–44	89	19.6
	45–49	44	9.7
	50–75	22	4.8
Sex	Male	206	45.3
	Female	249	54.7
Ethnicity	Oromo	378	83.1
	Amhara	68	14.9
	Tigre	4	0.87
	Others^a^	5	1.09
Occupation	Merchant	185	40.7
	Farmer	109	24
	Daily laborer	108	23.7
	Civil servant	33	7.3
	Other^a^	20	4.4
Highest education attended	Never attend school	147	32.3
	Primary (1–8)	269	59.1
	Secondary (9–12)	33	7.3
	TVET/Higher education	6	1.3
Residency	Urban	300	65.9
	Rural	155	34.1
Marital status	Married	280	61.5
	Single	50	11
	Widowed	29	6.4
	Divorced	69	15.2
	Separated	27	5.9
Number of children	1–2	187	41.1
	3–4	208	45.7
	5–6	60	13.2

Other^a^ occupation: self-employer, private employer

Other ethnic^a^: Gurage, Silte, etc

Precise count of 185 (40.7%) the study participants depended on income generated from business activity to run their life, followed by 109 (24%) agricultural products. Exactly 269 (59.1%) of the study participants had attended primary school. Out of 455 study participants, 300 (65.9%) of them were residents of urban areas. From 455 of the study participants, 280 (61.5%) were married and 208 (45.7%) of them had 3–4 children (Table 1).

### Partner notification practice of study participants

**Fig. 1:**
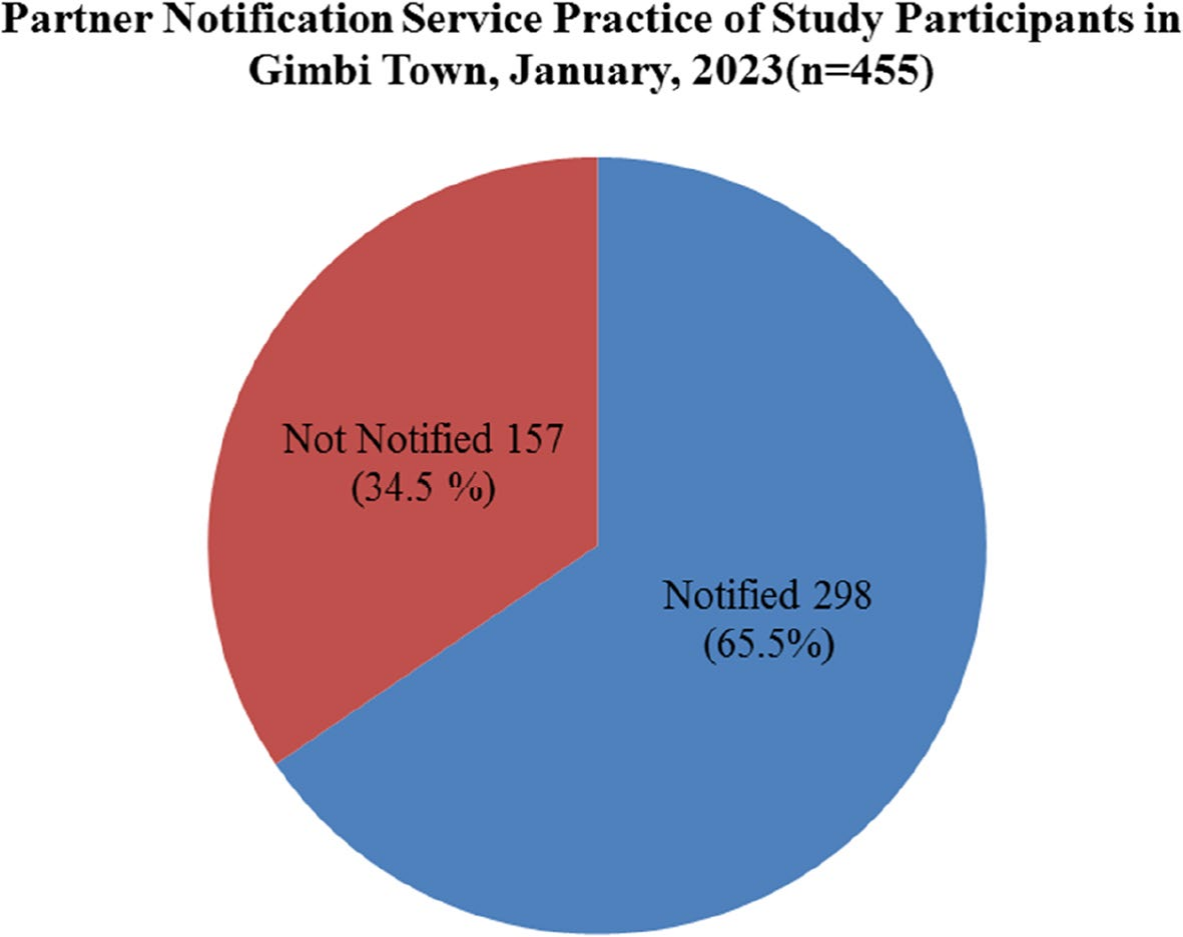
Partner Notification Practice of ART Clinic Attendants at Gimbi town Public Health Facilities, Gimbi (*n* = 455), January, 2023

From 455 study participants, 298(65.5%) of the study participants knew that their sexual partner was tested for HIV, while 63 (13.9%) of them did not test, and the remaining 94 (20.6%) did not know whether their partner was tested for HIV (Fig. 1). Of all HIV-tested sexual partners of the study participants, 215 (72.25%) were reported as positive and the remaining 38 (12.75%) were not yet known, while 45 (15%) were reported as negative for HIV.

Of the 455 study participants, 298 (65.5%) stated notifying their HIV status and referring their sexual partner. Subsequently, their respective sexual partners were tested for HIV after it was known that they might have been exposed to HIV/AIDS (Fig. 1). The remaining 157 (34.5%) were not notified of their sexual partner about potential exposure to HIV/AIDS for different reasons (Fig. 1).

According to this study, 440 (96.7%) of study participants responded that it is important to provide information about the potential exposure of their sexual partner due to their status to detect HIV among sexual partners to be beneficial from its health and economic advantage. The remaining 15 (3.3%) did not find its importance due to its outweighed disadvantage, such as loss of financial support and fear of isolation.

Regarding health system characteristics related to partner notification service, 408 (91.1%) of the facility’s clients can always get the health care staff, although relatively few of them claimed that they can only get in touch with the staff occasionally for HIV testing (Table 2). More than three-fourths (356, [79.5%]) of the study participants received all components of good counseling services for HIV testing in a convenient room to maintain their privacy. Out of all interviewed study participants, 448 (98.5%) of them reported that health professionals do not communicate their HIV status issue to anyone else without their permission, and 443 (97.4%) of them can access health facilities without physical difficulty for HIV testing services.

**Table 2:** Health System Characteristics for Partner Notification Service Utilization Study for HIV testing in Public Health Facilities Gimbi town, January, 2023 (*n* = 455)

Characteristics	Frequency		Percent
Availability of health workers for HIV testing	Always	415	91.2
	Sometimes	40	8.8
Good counseling	Yes	358	78.7
	No	97	21.3
Confidentiality break	Yes	7	1.5
	No	448	98.5
Inaccessibility of health facility	Yes	12	2.6
	No	443	97.4
Discrimination from staff	Yes	15	3.3
	No	440	96.7
Unavailability of HTS	Yes	6	1.3
	No	449	98.7
Adherence	Good	348	76.5
	Poor	107	23.5

*HTS* Human Immunodeficiency Virus Testing Service

Out of 455 study participants, 330 (72.5%) of them are taking ART medication according to instructions given to them to enhance adherence (Table 2). Exactly 449 (98.7%) of the study participants reported that they did not experience discrimination from health professionals working in ART clinics or the unavailability of Human Immunodeficiency Virus Testing Service (HTS).

According to information gathered from study participants, a substantial percentage of them stated that the use of partner notification services in the study area could be influenced by psychological factors. These factors were fear of intimate sexual partner violence, fear of losing a sexual partner’s support, divorce, stigma, accusation of infidelity, fear of isolation, intimate partner verbal abuse, discussion about HIV with partner, having depression, and intimate partner emotional abuse.

Based on the study participants’ responses, 298 (64.6%) of them did not have a fear of intimate partner violence and 305 (67%) did not worry about the dissolution of their marriage (Table 3). The majority, 334 (73.4%), of the study participants also did not have fear of stigma and discrimination due to their HIV serostatus. From all study participants, 267(58.7%) of them reported that they did not fear loss of support from their sexual partner to notify them of potential exposure to HIV infection.

**Table 3:** Psychological Characteristics for Study of Partner Notification Service Utilization in Gimbi Town Public Health Facilities, January 2023 (*n* = 455)

Characteristics	Category	Frequency	Percent
Fear of intimate partner violence	Yes	157	34.5
	No	298	65.5
Fear of loss of support	Yes	188	41.3
	No	267	58.7
Fear of divorce	Yes	150	33
	No	305	67
Fear of stigma	Yes	121	26.6
	No	334	73.4
Fear of accusation of infidelity	Yes	89	19.6
	No	366	80.4
Fear of isolation	Yes	110	75.8
	No	345	24.2
Intimacy to sex partner	Yes	331	72.7
	No	124	27.3
Discussion about HIV with sexual partner	Yes	325	71.4
	No	130	28.6
Depression	Yes	137	30.1
	No	318	69.9

### Factors associated with Partner Notification Service Utilization

According to this study, factors such as depression, fear of loss of economic support, fear of intimate partner violence and place of residency were found to have statistically significant associations with partner notification service utilization in multivariable logistic regression analysis. Their AOR was calculated by moving candidate variables showing a significant association with the outcome variable in the bivariable analysis to the multivariable logistic regression model after checking for multicollinearity using the variance inflation factor.

The probability of partner notification among study participants with any degree of depression was AOR: 0.12 (95% CI: 0.07, 0.20) (Table 4). This shows that study participants with any degree of depression were 88% less likely to notify their sexual partner than those who did not have depression.

**Table 4:** Multivariable Analysis of Variables Associated with Partner Notification Service Utilization in Public Health Facilities in Gimbi Town, January 2023 (*n* = 455)

Variables *n* = 455	Variable Category	Partner Notification Service utilization		COR (95% CI)	AOR (95%CI)	*P*-value
		Yes	No			
Depression	Yes	97	128	0.1(0.68–0.17)	**0.12(0.07–0.20)**	**0.001**
	No	201	29	1		
Intimacy to sex partner	Yes	65	19	2.01(1.16–3.5)	1.09(0.53–2.24)	0.81
	No	233	138	1		
Sex	Male	142	64	1		
	Female	156	93	0.75(0.51–1.11)	0.81(0.477–1.37)	0.43
Partner status	Positive	250	119	1		
	Negative	48	38	1.66(1.03–2.68)	1.37(0.74–2.55)	0.31
Discussion about HIV	Yes	93	37	1.47(0.94–2.29)	2.02(0.85–4.80)	0.11
	No	205	120	1		
Fear of support loss	Yes	78	108	0.15(0.09–0.23)	**0.24(0.14–0.40)**	**0.001**
	No	220	47	1		
Fear of stigma	Yes	72	49	0.7(0.45–1.07)	94(0.55–1.60)	0.83
	No	226	108	1		
Fear of infidelity accusation	Yes	133	94	0.54(0.365–0.8)	0.62(0.37–1.06)	0.08
	No	165	63	1		
Intimate partner violence	Yes	176	122	0.41(0.26–0.64)	**0.55 (0.31–0.97)**	**0.042**
	No	122	35	1		
Fear of isolation	Yes	64	46	0.65(0.42–1.02)	0.46(0.2–1.04)	0.06
	No	234	111	1		
Residency	Urban	111	44	1.52(1.0–2.3)	**2.21(1.27–3.83)**	**0.005**
	Rural	187	113	1		

*HIV* Human immunodeficiency virus, 1 reference

In study participants who fear loss of economic support from their sexual partners, the odds of utilizing partner notification service for HIV testing is AOR: 0.24 (95% CI: 0.14, 0.40) (Table 4). This statement implies that study participants with fear of loss of economic support from their sexual partner were 76% less likely to notify their sexual partner than study participants who do not worry about loss of economic support from their sexual partner. The other factor that showed a statistically significant association with the partner notification service utilization factor was place of residency. The probability of partner notification service utilization among study participants who live in urban areas was AOR: 2.21 (95% CI: 1.27, 3.83) (Table 4). This indicates that study participants who live in urban areas were 2.21 times more likely to notify their sexual partners than participants who live in rural areas.

In study participants who had experienced fear of intimate partner violence, the odds of notifying their sexual partner was AOR: 0.55 (95% CI: 0.31, 0.97) (Table 4) according to this study. This implies that study participants who fear intimate partner violence were 45% less likely to notify their sexual partner about potential exposure to HIV infection than those who do not.

### Qualitative result

A total of twelve HIV-positive adherence supporters from the three health facilities participated in an in-depth interview (8 females and 4 males), and six (2 female and 4 male) ART providers from each facility participated in a key informant interview.

All key informant interviews and in-depth interview participants were married and their ages ranged from 30 to 44 years old. All in-depth interview participants had attended their school up to grade 12. Regarding key informant interviews, all were health care professionals with an academic level of bachelor degree in nursing who were working in selected health facilities in the ART unit and were ART providers.

All in-depth interview participants explained that an individual with HIV infection should bring his/her sexual partner for HIV testing by informing his/her potential exposure to HIV/AIDS secondary to his/her sero status. The response from the key informant interview indicated that they are currently providing targeted HIV testing services, such as index case testing and partner notification services for HIV testing, even though there are a number of challenges to do so. The majority of the study participants expressed that a partner notification service for HIV testing is crucial to maximize yield and control its transmission. According to the response of study participants from all selected health facilities for the study, there was a conducive condition to the utilization of partner notification services for HIV testing, as they are providing targeted HIV testing services such as index case testing, partner notification services, respondent-driven HIV testing and key population testing in an easily accessible manner with strong emphasis.

“*It is important that all HIV positive clients should disclose their status to their respective sexual partner and bring them for testing for better outcome”.*

A 35-year-old male from in-depth interview participants.

Sexual contact tracing has been implemented at all selected health facilities by different strategies, such as requesting clients to provide the name of a new sexual partner and encouraging them to bring for HIV testing with the aim of addressing individuals with potential exposure to HIV early in the course of their infection.

Health education and family-focused HIV prevention strategies should be provided for every client visit to enhance this service as a recommendation. Stakeholders should actively engage in capacity building for providers and find other easy strategies in addressing populations with undiagnosed HIV status through this partner notification service (Table 5).

*“Since many people are unaware of the impact of unprotected sexual contact, HIV counseling and health education should be provided intensively to encourage them to notify sexual contacts because the success of this service depends on how well clients understand the case.”*

**Table 5:** Codes, Categories and Thematic Classification of Qualitative Data Analysis

Theme	Subtheme	Codes
Perceived Availability of Partner Notification Service	Health Care Service	Targeted HIV testing, Sexual contact tracing, Family based HIV testing, Increased access to HIV testing
Challenges of Partner Notification Service Utilization	Sociocultural Factors	Stigma, Discrimination, Isolation, Divorce Loss of economic support
Barriers of Partner Notification Service Utilization	Psychological Factors	Fear of intimate partner violence Poor discussion Fear of assassination attempt

A 38-year-old male key informant interview participant.

Study participants reported that a significant number of clients do not have an interest in notifying the potential exposure to HIV infection for their sexual partner because of the higher chance of becoming stigmatized and discriminated secondary to their HIV status in case of a confidentiality break.

… “*Since there is defamation in our society because of my HIV status, it is not necessary to disclose my status everyone else arbitrarily. So I should have to wait for a conducive condition to notify my partner selectively as much as I can.”*

A 30-year-old adherence supporter from in-depth interview participants.

According to this study, isolation, divorce and loss of economic support are considerable major challenges to partner notification utilization. The chance of marriage dissolution, isolation from society and loss of economic support, especially among female index clients, was high.

“*There was a case that one index client has been divorced and lost her support because of notifying her partner for HIV testing considering she might be a source of the infection after knowing his status which indicates discordant”.*

32-year-old male ART service providers from key informant interview.

Key informant interviews and in-depth interviews participants explain that most index clients, especially females, mostly fear intimate partner violence to notify their partner. Most in-depth interview participants and key informant interviews suggest that a significant number of index clients who attempted to notify their sexual partner faced isolation from their surrounding community, separated from their sexual partner and finally lost support from their sexual partner, especially female index clients. Intimate sexual partner violence and assassination warnings from their sexual partner because of notification of HIV status are of the reason clients hesitate to notify their partner.

“…... *there was one case when her partner beaten and hurt her when she attempted to notify him and provide HIV self-test*”.

30-year-old female ART service provider from key informant interviews.

## Discussion

The study was conducted to assess the magnitude of PNS utilization and associated factors among HIV-positive clients attending ART clinics at public health facilities in Gimbi town. The study identified that the majority (65.5% at 95% CI 61.3–69.7) of study participants had notified their sexual partner about the potential exposure of their partner to HIV infection because of their HIV seropositive status. This magnitude of partner notification utilization for HIV testing is less than evidence of partner notification service utilization in Kenya (77%), Cote Divore (97.2%) and Utah (79%) but higher than a study conducted in Tanzania (56.6%) and Seiralione (0.4%) [7, 11, 14, 26]. This discrepancy might be due to differences in socioeconomic conditions and health care systems under which the populations go. The study also reveals that a considerable number of sexual partners of HIV-positive clients were not aware of their potential exposure to HIV infection because of their sexual partner, which makes it below the WHO recommendation, as it states that all HIV-positive clients should notify their respective sexual partner about potential exposure to HIV infection using different approaches of partner notification [1].

The possible explanation for the higher magnitude of utilization than this study might be due to the study design employed in Utah, which was a two-year prospective follow-up study. As such, the type of data used for the study in the case of Kenya was secondary data from index clients’ medical records [7, 14]. In the case of Cote Divore, the study was conducted primarily to compare the efficacy of the four partner notification strategies, while this study used primary data collected from study participants without considering the strategies of partner notification they used using a cross-sectional study design [26].

The lower magnitude may also be due to the involvement of data collectors in the process of partner notification service delivery, as in the case of Tanzania [13]. In the case of the study in Sieralione, it might be contribution of study design employed as such it was retrospective cohort from secondary data as well as it only focus on tested partners as the main measure of partner notification utilization [11]. In this study, data collectors have no role in the process of delivering the service rather than collecting primary data from clients.

The results from qualitative data supported the findings from quantitative data, as the majority of the respondents expressed that partner notification service utilization for HIV testing is very important for yield maximization and control of the disease. A qualitative study from Malawi indicated that a significant number of index clients explained that couples counseling as a way to expand HIV testing through partner notification by providing accurate information for index clients [27]. This is comparable with this study result that intensive counseling and health education provision for index clients can increase partner notification service for HIV testing because counseling can build confidence in how to manage the reaction of sexual partners and create understanding on how to approach their sexual partner [27].

In this study, having any degree of depression decreased the probability of using partner notification for HIV testing by 88% compared with those free of depression. This finding is supported by a study performed in Spain by a systematic review of qualitatively conducted studies on similar issues in several countries [22]. This might be secondary to the fact that most clients with HIV infection are vulnerable to common feelings such as disappointment, sadness, fear, despair and loneliness, which can lead to developing depression.

This study noted that living in urban areas could increase the probability of notifying about potential exposure to HIV infection of sexual partners of HIV-positive clients to suggest HIV testing by 2.21-fold. This finding is in line with a related study conducted in Ethiopia to identify factors associated with HIV testing uptake among young females that identified living in rural areas as a factor that can decrease HIV testing uptake [28]. This may be due to the easy accessibility and availability of HIV testing services and information about HIV prevention and control in urban areas.

Fear of loss of economic support from sexual partners is also one of the factors that can negatively affect the utilization of partner notification services for HIV testing according to this study finding. According to this study, fear of loss of economic support from sexual partners can decrease partner notification utilization by 76%.

This statement is supported by the results of the qualitative part of this study, as the study participants expressed that fear of loss of economic support from sexual partners is among the barriers to partner notification utilization. This finding is supported by evidence from a related but not similar study conducted in Ethiopia to assess the HIV status disclosure of pregnant women to their sexual partners at Hawassa University Referral Hospital [23]. This comparability of both studies might be because the majority of the study participants of both studies were from urban areas where HIV/AIDS information, care and support services are accessible, the study design employed was similar, and the majority of PLHIV on ART follow-up were females who may be dependent on their sexual partner and the health care system from which both communities served.

The odds of partner notification utilization for HIV testing decreased among clients with fear of intimate partner violence by 45%. This statement is also supported by the qualitative results of this study. The finding was in line with studies conducted in Kenya, Cameron and the USA on similar issues [5, 15, 29]. This correspondence of the study results may be due to the similarity of the approach of providing the service in all settings despite contextual variation in the study area and methodological differences among the studies.

The results from the qualitative part of this study indicate that psychological factors such as fear of stigma, discrimination and fear of divorce were factors that can decrease the chance of utilizing partner notification services for HIV testing among index clients. This idea was supported by the findings of a study conducted in Spain by a systematic review of different articles conducted qualitatively [22]. This correspondence of findings may be due to PLHIV having common feelings, such as a sense of hopelessness, loneliness and, in some communities, mislabeling of HIV infection, as it is the result of the mis-behavioral acts of clients. However, these factors had no statistical association with the outcome variables in the quantitative part of this study.

This study found that a considerable number of PLHIV in the study area are not utilizing partner notification services for HIV testing due to fear of adverse outcomes from their respective sexual partners after notification. Therefore, to address the remaining portion of PLHIV with partner notification services for HIV testing, the provision of psychological support, economic strengthening for PLHIV and the expansion of community-based HIV care and support may be needed from local health system structures and stakeholders. Overall, the study identified poor partner notification service utilization when compared to WHO recommendation due to barriers and challenges differentiated to have an association in both quantitative and qualitative finding.

### Strength and limitation

Qualitative and quantitative methods were used for this study. These methods reinforce each other in achieving the study goal. The study was conducted at the health facility level. Therefore, the study results may not be generalizable to all people with HIV infection in the entire community. Some of the information may differ from the actual one due to social desirability during quantitative data collection.

## Conclusion and recommendations

This study showed that most index clients have been notified their sexual partner about potential exposure to HIV infection and suggest testing for it, but a considerable number of index clients still did not utilize partner notification for HIV testing. Major reasons for index clients not notifying their sexual contact were fear of intimate sexual partner violence, loss of economic support and depression. Living in urban areas was identified as a facilitator of partner notification service utilization in the study area.

To address the remaining portion of HIV-exposed sexual partners of PLHIV in the study area, the Gimbi Town Health Office, religious leaders and donors should create a forum with People Living with HIV Association on how to decrease fear of intimate partner violence and strengthen positive living attitudes in PLHIV and social ties. The PLHIV Association should implement economic strengthening activities in collaboration with local implementing organizations and the Gimbi Town Labor and Skills Office.

Health care providers should routinely screen index clients for the presence of depression; actively sustain their counseling effort and provide strong attention for clients coming from rural areas.

## Data Availability

All statistical data used in this study are available from the corresponding author upon reasonable request.

## Abbreviations

AIDS: Acquired immunodeficiency syndrome
AOR: Adjusted odds ratio
CI: Confidence interval
HCT: Human immunodeficiency virus counseling and testing
HIV: Human immunodeficiency virus
HTS: Human immunodeficiency virus testing service
ICT: Index case testing
IPV: Intimate partner violence
PLHIV: People living with human immunodeficiency virus
PLHIVA: People living with human immunodeficiency virus association
